# Different patterns of myopia prevalence and progression between internal migrant and local resident school children in Shanghai, China: a 2-year cohort study

**DOI:** 10.1186/s12886-018-0716-3

**Published:** 2018-02-23

**Authors:** Yingyan Ma, Senlin Lin, Jianfeng Zhu, Xun Xu, Lina Lu, Rong Zhao, Huijuan Zhao, Qiangqiang Li, Zhiyuan Hou, Xiangui He, Haidong Zou

**Affiliations:** 1 Department of Preventative Ophthalmology, Shanghai Eye Disease Prevention and Treatment Center, Shanghai Eye Hospital, No. 380 Kangding Road, Shanghai, China; 2 Department of Ophthalmology, Shanghai General Hospital, Shanghai Jiao Tong University, No.100, Haining Road, Shanghai, China; 3 Department of Social Medicine, School of Public Health, National Key Laboratory of Health Technology Assessment (National Health and Family Planning Commission), Collaborative Innovation Center of Social Risks Governance in Health, Fudan University, No. 138 Yixueyuan Road, Shanghai, China; 4 Shanghai Shen Kang Hospital Development Center, No.2 Kangding Road, Shanghai, China; 5 Baoshan Center for Disease Prevention and Control, No. 158 Yueming Road, Shanghai, China

**Keywords:** Migrant children, Myopia, Progression, Spherical equivalent refraction, Axial length

## Abstract

**Background:**

In 2010, there were ~ 36 million migrant children under 18 y old in China. This study compared patterns of myopia prevalence and progression between migrant and resident children.

**Methods:**

Eight hundred forty-two migrant children from 2 migrant schools and 1081 from 2 local schools in Baoshan District, Shanghai, were randomly chosen. Baseline measurements were taken on children in grades one through four, and children in grades one and two were followed for 2 y. The children underwent comprehensive ophthalmic examinations, including cycloplegic refraction and axial length. The average time per week spent on homework and outdoor activities were investigated.

**Results:**

Migrant children in grades one and two showed a lower myopia prevalence than resident children; however, from grades three to four, the prevalence accelerated and exceeded that of residents. In the follow-up, the myopia incidence did not significantly change from grades one to two in resident children but was significantly higher in grade two in migrant children. Correspondingly, for migrant children, increased progression of refraction and axial length was observed; however, it decreased in resident children. The average time spent on homework increased from grades two to three in parallel with the acceleration of myopia prevalence for migrant children; however, the time spent outdoors did not correspondingly change.

**Conclusion:**

The patterns of myopia prevalence and progression are different between migrant and non-migrant children. The acceleration of myopia in migrant children might be a result of a change in their environment, such as intensive education pressure.

## Background

With rapid economic development and urbanization during the past few decades in China, a large number of adults and their children have moved from rural to urban areas, constituting 273.95 million internal migrants by 2014 [1, 2]. The 2010 National Population Census revealed ~ 36 million migrant children in China, comprising 12.5% of the population under 18 y old [3]. In addition to the discrepancy in educational opportunities, migrant children are ineligible for other urban social welfare programs, such as public health care and financial assistance [4]. The restrictions on access to urban social welfare, in addition to relatively lower economic and sanitary conditions, make migrant children more vulnerable than urban residents to diseases [1, 5, 6]. Although many studies have focused on the mental health, nutritional problems, vaccination coverage, and communicable diseases of these children [1, 5, 6], very few have been conducted in China on their ocular health [7, 8].

The prevalence of myopia is especially high in children of East-Asian ethnicity [9]. Among 15-y-old children, the prevalence is highest in Singapore (86.2%), followed by Taiwan (80%), Hong Kong (78.2%), and mainland China (59%) [9]. In general, children from urban areas demonstrated an obviously higher prevalence for myopia than those from rural settings [9–11]. In mainland China’s Shunyi District, a rural area, myopia prevalence in 15-y-olds was 36.7% for boys and 55% for girls, and was 73.4% for boys and 83.2% for girls in Guanzhou, an urban city [12, 13]. Migrants comprise a special group of children that are usually born in rural areas, but move with their parents to live and study in urban cities, providing a natural condition by which to explore the influence of environmental changes on myopia within the same group of children. Whether their myopia prevalence and progression are as high as that of urban children or are comparatively similar to those of rural children is not clear.

There have been only two cross-sectional studies on myopia prevalence among migrant children in China. One study by He et al. [7] reported that uncorrected refractive error, especially myopia, was the major cause of visual impairment in migrant children. Another study reported that the prevalence of myopia in primary school was 30.3% in migrant children, lower than that in resident children (33.9%), suggesting a protective effect of migration [8]. Nevertheless, the prevalence of myopia changed rapidly as grade level increased, especially during the primary school years; therefore, the cross-sectional research cannot capture the progression of myopia, and cannot identify whether myopia incidence and progression are different between migrants and residents. Longitudinal studies are necessary to understand the patterns of myopia incidence and progression between migrant and resident children.

To determine myopia problems in migrant children in urban China, the present study, through a follow-up of primary school students in both migrant and local schools for 2 y, compared changes in the patterns of refraction and refractive components between the internal migrant children and their local resident counterparts. This study will also provide valuable suggestions for alleviating the disease burden of myopia in this normally fragile population.

## Methods

### Study settings and participants

Shanghai, one of the most developed regions in China, was the site for our study. According to *Shanghai Statistical Yearbook*, the number of internal migrants had increased dramatically from 3.87 million in 2000 to 11.22 million in 2010, accounting for nearly one-half of the total population of this city [14]. Children of these migrants also accounted for ~ 46.2% of all children in Shanghai in 2010 [15]. Baoshan District, located north of Shanghai, was one of the largest migrant population import areas [16]. In 2010, the average gross domestic product per capita was 54,657 RMB in Baoshan District, lower than that in Shanghai (76,074 RMB) [14, 16].

**Fig. 1:**
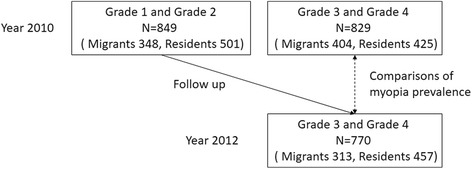
Flow chart of the study design

The increasing migrant population has imposed great pressure on the local urban education system, and as a result, any migrant children without a permanent registered urban residence (Hukou) do not have access to normal public schools. Consequently, most migrant children are enrolled in private schools, which were specifically established for migrants (hereinafter refer to “migrant schools”), to meet the increasing educational demands; however, with much less support from government, those migrant schools were usually without proper equipment and could employ only low-quality teachers [17, 18]. In the present study, two private primary schools, established specifically for internal migrant children and two public primary schools mainly for local resident children (hereinafter refer to “local schools”), were randomly selected in Baoshan District, and children in grades one to four from these schools were included in the study. Grade five students were not included in the study because of the extremely low participation rate most likely from intensive study pressure on the students to enter junior high school. Our study chose the longitudinal research design and followed the students for 2 y, with the first visits from May 2010 to April 2011 and the second visits from May 2012 to April 2013. The order by which each of the four schools was examined was the same during the first and second visits to ensure a 2-y gap between visits to each school. Figure 1 displays the study design. Considering the length of time spent in primary school in Shanghai (five grades), only children from grades one to two were chosen and followed for 2 y because those in grades three and four graduated after 2 y. The cluster random sampling technique was used for selecting the students. In the 2010 baseline measurements, 1923 students from grades one through four from four schools were included. Children with severe ocular diseases other than refractive error, such as congenital cataract, and children who would not cooperate with the examinations were excluded from the study.

### Examinations

A research group consisting of one ophthalmologist, five optometrists, and two public health doctors was involved in the examinations performed in the schools. Before the formal examinations, members of the research group were trained according to exact protocols and were tested for eligibility of their specific examination item. Children underwent tests for visual acuity (uncorrected) (Standard Logarithmic Visual Acuity E Chart, 5 m), slit lamp examinations, measurements of intraocular pressure by noncontact tonometer, tests for cycloplegia, tests for cycloplegic autorefraction, measurements of corneal curvature radius (CR) using the KR-8800 table-mounted autorefractor (Topcon, Tokyo, Japan), measurements of axial length (AL) using an IOLMaster (v. 5.02, Carl Zeiss Meditec, Oberkochen, Germany), and tests for best corrected visual acuity. Cycloplegia was induced by administering 1 drop 0.5% tropicamide every 5 min and repeating five times. Pupil size and light reflex were examined 20 min after the last drop of tropicamide, and if the pupil dilated ≥6 mm and light reflex was absent, cycloplegia was deemed complete. The average reading of three consecutive measurements of refraction and AL were calculated for data analyses. The ophthalmologist examined children under a slit lamp and determined whether a child was suitable for cycloplegia. The optometrists manipulated the measurement for intraocular pressure, autorefraction, AL, and CR. Any children with uncorrected visual acuity lower than 20/25 in either eye were given subjective optometry to obtain the best corrected visual acuity.

To explore the reason for the changes in the patterns of myopia prevalence with school grade between migrant and resident children, we did a quick assessment in 2010 using questionnaires on the time the children spent on homework and outdoor activities. The questionnaires were distributed to students to record the times for these activities over the previous week with the help of their parents.

### Statistical analyses

We used Epidata 3.1 to create a database for recording all measurements. All data were independently entered twice by two research assistants, and all discrepancies were adjudicated. Statistical analyses were conducted using SAS v. 9.3 (SAS Institute, Cary, NC, USA).

Spherical equivalent refraction (SE) was calculated as spherical diopters + 0.5* cylinder diopters. Myopia is defined as SE ≤ − 0.5 diopters (D) in the right eye. Because the right eye is usually largely correlated with the left eye, measurements of the right eye were included in the analyses. The incidence of myopia was defined as not myopic at baseline (2010) and myopic 2 y later in 2012. The progress of SE was calculated as the student’s SE in 2010 minus his/her SE in 2012. A positive value reflected progression into a more myopic refraction. Similarly, the progress of AL was calculated as the student’s AL in 2012 minus his/her AL in 2010, and the larger the AL value, the higher the myopic refraction.

Chi-squared and t-tests were applied for comparing basic characteristics, myopia prevalence, and refractive status between migrant and resident children at baseline (2010). Multivariate logistic regressions with and without interaction terms were performed to explore the potential risk factors for a 2-y myopia incidence. The first model comprised the main effects of the independent variables, while the second comprised the additional interaction effects between migration and grade. Furthermore, to analyze changes in refractive status, we applied a linear regression to two continuous indicators—2-y SE progression and 2-y AL increase—as dependent variables. Just as in the logistic regression, two models with and without interaction terms were applied for each indicator, respectively. Statistical significance was defined as *p* < .05 (two tailed).

## Results

### Inclusion of students in the baseline and follow-up

There were 842 students from grades one through four in the two migrant schools, and 1081 students from grades one through four in the two local schools for which baseline data were to be compiled. In the baseline analyses, after excluding those without a written informed consent, those who were uncooperative with the examinations, and those who suffered from severe ocular diseases other than refractive error, 752 (89.3%) children from migrant schools and 926 (85.7%) from local schools were included. Among the 849 (348 for migrants and 501 for residents) students in grades one and two included in the baseline data, 313 (89.9%) migrant students with a mean age of 7.89 y (SD = 0.88) and 457 (91.2%) students of local schools with a mean age of 7.54 y (SD = 0.75) were followed for 2 y, corresponding to the students in grades three and four in 2012. The average followup time for the current population was ~ 24 months.

### Prevalence of myopia between 752 children from the migrant schools and 926 children from local schools in the baseline

**Fig. 2:**
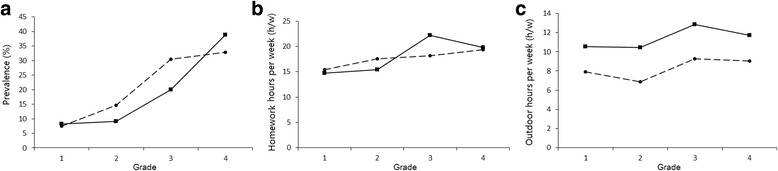
Prevalence of myopia and hours spent per week between migrant and local children at baseline. Prevalence of myopia (**a**) and hours spent on homework (**b**) and outdoor activities (**c**) per week in 752 migrant children and 926 resident children of grade 1 to 4 at Year 2010 were shown in Fig. 2. Solid line with square stands for migrant children; dashed line with circle stands for resident children

In the baseline, the myopia prevalence changed from grades one through four between the migrant and resident children. For children in grade one, the prevalence of myopia was similar between the two populations (7.42% for residents and 8.15% for migrants, *P* = .78). For the children from local schools, the myopia prevalence increased steadily from grades one through four; however, the migrant children showed an accelerated prevalence after grade two, resulting in a prevalence of myopia higher than that in resident children by grade four (Fig. 2a).

### Comparison of the 2-y myopia incidence, progression, and axial length progression among 313 migrant children and 457 children from local schools who were followed

**Fig. 3:**
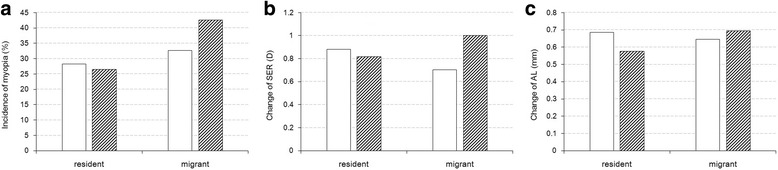
Myopia incidence (**a**), change of SE (**b**) and AL (**c**) between resident and migrant children. Blank rectangles stand for grade 1 and rectangles with oblique line stand for grade 2. SE, spherical equivalent refraction; AL, axial length

For those included in the 2-y cohort, in the baseline, the percentage of males was higher in migrant children, and the refractive status was more myopic for parents of children from local schools, while the refractive status and components were not statistically different between the two populations (Table 1). During the 2-y follow-up, the incidence of myopia did not change significantly from grades one (28.2%) to two (26.4%) for the resident children (*P* = .6786); however, the incidence of myopia in grade one migrant children (32.7%) was less than that in grade two migrant children (42.6%) (*P* = .0828, Fig. 3a). Accordingly, increased progression of refraction and axial length toward myopia were observed in migrant children from grades one to two (*p* = .0012 and *p* = 0.2211, respectively), and the progressions decreased in resident children as grades increased (*p* = .4471 and *p* = .0026, respectively) (Figs. 3b, c).

**Table 1:** Comparison of baseline characteristics between 457 resident and 313 migrant children who completed 2-year follow-up

		Resident *n* = 457	Migrant *n* = 313	Chi-square/t value	*P* Value
Grade, No (%)	1	227 (49.7)	159 (50.8)	0.0944	0.7587
	2	230 (50.3)	154 (49.2)		
Gender, No (%)	male	233 (51.0)	184 (58.8)	4.5538	0.0328
	female	224 (49.0)	129 (41.2)		
Age, years (SD)		7.54 (0.75)	7.89 (0.88)	5.926	< 0.0001
Parental Myopia, No (%)	0	123 (26.9)	201 (64.2)	107.9569	< 0.0001
	> = 1	87 (19.0)	21 (6.7)		
	unknown	247 (54.1)	91 (29.1)		
Prevalence of Myopia, No (%)		51 (11.2)	25 (8.0)	2.1018	0.1471
SE, Mean (95% CI), D		0.59 (0.50–0.68)	0.46 (0.35–0.57)	1.89	0.0588
AL, Mean (95% CI), mm		22.97 (22.90–23.04)	22.94 (22.85–23.02)	0.62	0.5352

*SE* spherical equivalent refraction, *AL* axial length

### Factors associated with myopia incidence and progression of SE and AL

Logistic regression of myopia incidence is listed in Table 2. For local school children, the incidence of myopia was not significantly associated with grade level (aOR = 0.780, *p* = .4048, model 2); however, for migrant children, students in grade two showed a significantly higher incidence of myopia than those in grade one (aOR_interaction_ = 2.525, *p* = .033) (Table 2, model 2). This pattern change was further confirmed by the changes in SE and AL in the migrants and the children in local schools (Table 3). For the migrant children, the students in grade two showed an increased change of SE compared with those in grade one over 2 y (*p* = .0016). For the children of local schools, the students in grade two showed a decrease in the change in axial length elongation compared with the students in grade one (*p* < .001); however, for the migrant children, the students in grade two showed no significant changes in axial length progression compared with those in grade one (*p* = .2406).

**Table 2:** Multivariate logistic analysis for myopia incidence (Only including children who were not myopia at baseline)

Variable		MODEL 1	MODEL 2
Migrant		1.162	0.72
		(0.728–1.855)	(0.379–1.368)
Grade 2		1.205	0.780
		(0.788–1.843)	(0.436–1.398)
Grade2*Migrant		–	2.525
			(1.078–5.916)*
Female		1.146	1.198
		(0.724–1.813)	(0.755–1.902)
Parental Myopia	0	REF	REF
	> = 1	1.139	1.131
		(0.575–2.254)	(0.570–2.242)
	Unknown	1.179	1.175
		(0.721–1.928)	(0.718–1.921)
Baseline SE		0.017	0.017
		(0.009–0.033)‡	(0.009–0.032)‡
Baseline AL		0.718	0.721
		(0.503–1.025)	(0.505–1.030)

Odds ratios and 95% confidence intervals were shown

Significance level: *p < 0.05, ‡*p* < 0.001

*SE* spherical equivalent refraction, *AL* axial length

**Table 3:** Multivariate linear regression analyses for progression of SE and AL in 2 years

		Progression of SE		Progression of AL	
		MODEL 1	MODEL 2	MODEL 1	MODEL 2
Variable					
Migrant		0.0665	−0.1394	0.0637	−0.0276
		(−0.0626 to 0.1957)	(−0.3122 to 0.0333)	(0.0094 to 0.118)*	(−0.1003 to 0.0451)
Grade 2		0.0463	−0.1222	−0.0645	− 0.1391
		(− 0.0716 to 0.1643)	(− 0.2727 to 0.0284)	(− 0.1141 to − 0.0149)*	(− 0.2023 to − 0.0758)‡
Grade2*Migrant		–	0.4194	–	0.1855
			(0.1835–0.6553)‡		(0.0864 to 0.2846)†
Female		0.2257	0.2342	0.057	0.0609
		(0.0995 to 0.3520)‡	(0.1089 to 0.3596)‡	(0.0039 to 0.1101)*	(0.0082 to 0.1135)*
Parental Myopia	0	REF	REF	REF	REF
	> = 1	0.3175	0.3261	0.1491	0.1529
		(0.1294 to 0.5055)‡	(0.1395 to 0.5127)‡	(0.0701 to 0.2281)†	(0.0746 to 0.2313)†
	Unknown	0.1644	0.1741	0.0934	0.0975
		(0.0311 to 0.2978)*	(0.0417 to 0.3065)*	(0.0373 to 0.1495)*	(0.0419 to 0.1532)†
Baseline SE		−0.2549	−0.2566	−0.1480	−0.1487
		(−0.3279 to −0.1819)‡	(−0.3290 to − 0.1842)‡	(−0.1786 to − 0.1173)‡	(−0.1791 to − 0.1184)‡
Baseline AL		0.0029	0.003	−0.0233	−0.0233
		(−0.0952 to 0.101)	(−0.0944 to 0.1003)	(− 0.0645 to 0.0179)	(− 0.0642 to 0.0175)

Coefficients and 95% confidence intervals were shown

Significance level: *p < 0.05, †*p* < 0.01, ‡*p* < 0.001

*SE* spherical equivalent refraction, *AL* axial length

### Changes in the pattern of myopia prevalence from 2010 to 2012 between migrant children and children in local schools in grades three and four

**Fig. 4:**
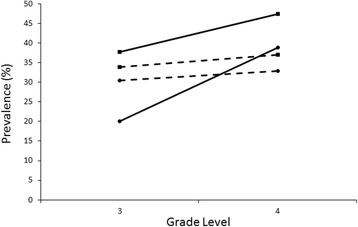
Change of myopia prevalence between 2010 and 2012 among migrant and resident children (Grade 3–4). Solid line with circle stands for myopia prevalence of migrant children in 2010; solid line with square stands for myopia prevalence of migrant children in 2012; dashed line with circle stands for myopia prevalence of resident children in 2010; dashed line with square stands for myopia prevalence of resident children in 2012

Additional analyses were conducted of myopia prevalence in students in grades three and four in 2010 (baseline) and those in 2012 (the same cohort of grades one and two in 2010) to explore whether time changes influenced the prevalence of myopia. Figure 4 shows the changes in myopia prevalence in students in grades three and four between 2010 and 2012. For the children in local schools, there were nearly no changes in myopia prevalence (chi-squared test, *p* = .4868 for grade three and *p* = .3350 for grade four); however, for the migrant children in grade four, the prevalence of myopia increased, although not significantly (*p* = .0003 for the grade three and *p* = .0972 for grade four children) (Fig. 4).

### Time spent on homework and outdoor activities among 752 children from migrant schools and 926 children from local schools recorded during the baseline visit

As recorded during the baseline visit, the average number of hours spent on homework steadily increased from grades one to four for the children in local schools; however, that sharply increased from grades two to three for the migrants, which most likely explains the parallel increases in myopia prevalence after grade two (Fig. 2a-c). In contrast, the time spent on outdoor activities showed no corresponding correlation with changes in myopia prevalence in the two populations.

## Discussion

The results of the present study showed changes in the pattern of myopia prevalence, incidence, and progression between the internal migrant children and the children in local schools. Unlike the resident children who showed a steady increase in myopia prevalence and a stable myopia incidence and progression, the migrant children presented an accelerated increase in myopia prevalence and refraction progression, resulting in an increased prevalence of myopia with time and revealing an unsatisfied status of myopia, which is worth attention.

In general, in cross-sectional studies, migrant children showed less myopia than resident children, and were even regarded as a protective factor [8]. In the present study, myopia prevalence was low in migrant children in the junior grade, but comparable or a litter higher in the senior grade (grade four) than that of the resident children. Lacking a follow-up study, the incidence and progression of myopia in migrant children remains unknown. Our study found that the crude 2-y incidence of myopia is even higher in migrant children than in resident children, as well as the 2-y progression of refraction and axial length. These migrant children, usually from less seriously–affected rural areas, presented a low prevalence of myopia in the junior grade; however, after being affected by the changing environment, showed a myopia prevalence similar to that of the local urban children in the senior grade. Our study captured this catch-up phenomenon, which could be associated with urban environmental factors.

A similar phenomenon was also observed in children of Chinese ethnicity who showed a relatively high prevalence of myopia if living in Singapore, Hong Kong, or Taiwan but a far lower prevalence if living in Australia [9]. In addition, the prevalence of myopia among second-generation (or higher) Indian immigrants in Singapore is higher than that in first-generation immigrants and in Indians living in India and Malaysia [19]. On the contrary, the Yunnan Minority Study [20] reported that people of different ethnicities living under the same environmental conditions shared a similar prevalence of myopia. The results of our study are consistent with those of previous studies and provide sufficient evidence for the importance of environmental factors on myopia incidence and progression.

Long periods of time at near work and short periods of time spent on outdoor activities are the two widely acknowledged environmental risk factors for myopia [21–23]. In the present study, the sudden increase in the time spent on homework might account for accelerated myopia prevalence, especially in migrant children in grades three and four (Fig. 4). Because migrant children come mostly from less-developed areas with a less-rigorous education system, they are usually less educated and have a poor foundation for learning in school. In addition, the migrant schools are usually incapable of recruiting high-level or experienced teachers and have less resources than local public schools; however, as grades increase, the education curriculum becomes more and more difficult; therefore, the migrant children must spend more time studying to catch up with their resident counterparts, resulting in an accelerated increase in the time spent on homework, as observed in our study. The present study did not observe a relationship between outdoor activities and myopia as did other studies conducted on Chinese school children [24, 25], most likely because the number of hours spent outdoors per week was generally low in the present population, which was not enough to have any effect on protection [21].

In addition, migrant children in grades three and four in 2012 presented a higher prevalence of myopia than those in 2010; however, an increase in myopia prevalence in resident children was not observed. The Beijing Myopia Progression Study and the Handan Offspring Myopia Study also reported an increasing prevalence of myopia in the younger generation and suggested a change in environmental factors as the reason [26, 27]. We presumed that in the present population, the study pressure increased with time in migrant children but remained unchanged in resident children. In addition to study pressure, other environmental risk factors existed, which harmed migrant children more than resident children. For example, with electronic devices usually under loose parental control, migrant children might be more involved in watching TV, playing on the computer, and using mobile phones or other electronic devices for entertainment than resident children. Those near work activities could aggravate the incidence or progression of myopia [28]. Hence, it is indicated that there are risk factors for myopia that change with time, making migrant children more vulnerable to myopia than resident children. To determine the reasons behind this, future studies must be conducted that collect detailed data on the risk factors of myopia in those migrant children.

Previous studies have reported that after the onset of myopia, the number of migrant children who wear corrective lenses was ~ 10% lower than that in resident children [29]. In addition, in those children with corrective lenses, 26.1% were inadequately corrected [7]. Not wearing or wearing inappropriate lenses could cause additional visual impairment in myopic children, influencing their study and living habits, reducing their quality of life, and affecting their healthy development [30–32]. Because myopia prevalence especially accelerates from grades three to four in migrant children, a relatively high demand for corrective lenses is anticipated during this period; therefore, reinforcing health education on the importance of wearing corrective lenses, distributing them free, or providing an allowance for prescribing or buying them could be valuable strategies to relieve the burden of reduced visual acuity caused by myopia in those migrant children. Information on myopia prevention methods, such as increasing the amount of time on outdoor activities and making myopia treatment available to slow its progression, such as the use of atropine and orthokeratology lenses, could be presented in the migrant schools to increase everyone’s knowledge about the disease.

### Study limitations

There were a few limitations to the present study that must be clarified. First, two migrant schools and two local schools were randomly selected. The school-based study design might have resulted in the participants not being representative of the objective population. Although all the children in migrant schools were migrant children who immigrated with their parents, not all children in local schools were resident children. Children with urban Hukou in the local schools could also have moved there from other parts of China; however, they could have lived in Shanghai for a relatively long period to establish local Hukou, thus their environmental risk factors would be similar to those of local resident children. Future studies with a larger sample size and with a design that includes a mixture of both local and migrant children within the same schools are needed to further clarify the phenomenon. Second, we did the followup examinations 2 y from baseline but did not conduct any annually. The followup period might have been too long to determine an accurate myopia incidence or progression in this young population because of their rapidly changing refractive status. Third, detailed information about myopia-related risk factors or information on migration, such as how long they had lived in the city and their original place of residence were not collected in the present study. Only reading and outdoor hours were assessed using a brief investigation; therefore, the reasons for the rapid increase in myopia incidence and progression in migrant children and the discrepancy between the two populations could not be determined. Finally, we used 0.5% tropicamide, which is a relatively weak cycloplegic reagent, in the study. Although evidence has proved that 0.5% tropicamide could be effective in measuring refraction in myopic children with dark pupils [33, 34], it could overestimate a child’s refraction toward myopia because of its relatively weak cycloplegic effect [35]; however, becuase the reagent was applied in both migrants and residents using the same standards during the baseline and followup, the differences from the true refraction could be consistent and would not influence the comparisons between migrants and residents.

## Conclusions

To the best of our knowledge, this study is the first to document different patterns of myopia incidence and progression among migrant children and resident children in China. Unlike their resident partners, who showed a slow and steady increase in myopia, the migrant children displayed accelerated myopia progression, resulting in rapid elongation of axial length. Although the crude prevalence of myopia could be lower in migrant children than in resident children during the primary school period, the incidence of myopia was higher in the migrants, making this population more at risk for developing myopia. Considering the relatively lower uses of health services in migrants, special attention should be paid to relieve the disease burden of myopia in this population.

## Abbreviations

AL: Axial length
CR: Corneal curvature radius
D: Diopters
SE: Spherical equivalent refraction
